# Identification and Characterization of miRNAs and lncRNAs Associated with Salinity Stress in Rice Panicles

**DOI:** 10.3390/ijms25158247

**Published:** 2024-07-28

**Authors:** Conghui Jiang, Yulong Wang, Yanan He, Yongbin Peng, Lixia Xie, Yaping Li, Wei Sun, Jinjun Zhou, Chongke Zheng, Xianzhi Xie

**Affiliations:** 1 Institute of Wetland Agriculture and Ecology, Shandong Academy of Agricultural Sciences, Jinan 250100, China; jiangch_sds@outlook.com (C.J.);; 2 Beijing Key Laboratory of Crop Genetic Improvement, College of Agronomy and Biotechnology, China Agricultural University, Beijing 100193, China; 3 Institute of Crop Germplasm Resources, Shandong Academy of Agricultural Sciences, Jinan 250100, China

**Keywords:** lncRNA, ceRNA, salinity stress, miRNA, rice

## Abstract

Salinity is a common abiotic stress that limits crop productivity. Although there is a wealth of evidence suggesting that miRNA and lncRNA play important roles in the response to salinity in rice seedlings and reproductive stages, the mechanism by which competing endogenous RNAs (ceRNAs) influence salt tolerance and yield in rice has been rarely reported. In this study, we conducted full whole-transcriptome sequencing of rice panicles during the reproductive period to clarify the role of ceRNAs in the salt stress response and yield. A total of 214 lncRNAs, 79 miRNAs, and 584 mRNAs were identified as differentially expressed RNAs under salt stress. Functional analysis indicates that they play important roles in GO terms such as response to stress, biosynthesis processes, abiotic stimuli, endogenous stimulus, and response to stimulus, as well as in KEGG pathways such as secondary metabolite biosynthesis, carotenoid biosynthesis, metabolic pathways, and phenylpropanoid biosynthesis. A ceRNA network comprising 95 lncRNA–miRNA–mRNA triplets was constructed. Two lncRNAs, MSTRG.51634.2 and MSTRG.48576.1, were predicted to bind to osa-miR172d-5p to regulate the expression of *OsMYB2* and *OsMADS63*, which have been reported to affect salt tolerance and yield, respectively. Three lncRNAs, MSTRG.30876.1, MSTRG.44567.1, and MSTRG.49308.1, may bind to osa-miR5487 to further regulate the expression of a stress protein (LOC_Os07g48460) and an aquaporin protein (LOC_Os02g51110) to regulate the salt stress response. This study is helpful for understanding the underlying molecular mechanisms of ceRNA that drive the response of rice to salt stress and provide new genetic resources for salt-resistant rice breeding.

## 1. Introduction

Salinity is a major abiotic stress factor that affects plant growth and crop production. Severe salt stress can cause ion imbalance, hyperosmotic stress, and oxidative damage in plant cells. Rice (*Oryza sativa* L.) is one of the most important staple food crops worldwide. However, it is a salt-sensitive cereal crop, especially at the early seedling and reproductive stages [1,2]. Salinity may cause a yield loss of 50–65% in rice crops, mainly due to decreases in the percentage of seed set, effective panicle number, and grain number per panicle [3]. Understanding the response of rice to salt stress can assist in breeding salt-tolerant cultivars.

To cope with environmental stress factors, rice has evolved sophisticated response mechanisms, such as stress perception, epigenetic modification, and the regulation of transcription and translation [4]. Numerous studies have examined the molecular mechanisms of the salt stress response in rice. However, most of these studies have primarily focused on the functional analysis of protein-coding genes [5]. In recent years, stress-responsive non-coding RNAs, such as microRNAs (miRNAs) and long non-coding RNAs (lncRNAs), have been identified as regulatory molecules that respond to abiotic or biotic stresses in plants [6,7]. MiRNAs, a type of small non-coding RNA, regulate gene expression at the post-transcriptional level [8]. For example, the overexpression of osa-MIR396c and osa-MIR393 in rice and *Arabidopsis* enhances their binding to target growth-regulating genes and other regulatory genes, leading to increased salinity sensitivity [9,10]. The OsmicroRNA156-IPA1 pathway may be involved in rice salinity responses, thereby affecting rice yield [11]. LncRNAs, a type of non-coding sequence with a transcription length exceeding 200 nt, rarely encode or cannot encode proteins due to the lack of an effective open reading frame. They can regulate the expression of protein-coding genes through *cis-* and *trans-*acting mechanisms [12,13]. The function of lncRNAs in salt stress has been examined in various crop plants. For example, the abundance of lncRNA973 is significantly increased in response to salt treatment in cotton, and it positively regulates salt tolerance [14]. The overexpression of lncRNA77580 enhances the drought tolerance of soybean but also leads to increased sensitivity to high salinity at the seedling stage [15]. The lncRNA TRABA suppresses the expression of the β-glucosidase-encoding gene *BGLU24* to promote salt tolerance in cotton [16]. Recent studies have reported that lncRNAs can function as competing endogenous RNAs (ceRNAs). In this mechanism, lncRNAs compete with messenger RNAs (mRNAs) to bind to miRNAs, thereby modulating the expression of target genes. The expression of lncRNA354 is decreased under salt stress, which weakens its binding to miR160b, a suppressor of GhARF17/18, thus upregulating miR160b and enhancing root development, thereby synergistically regulating salt tolerance in cotton [17]. Although a wealth of evidence suggests that miRNA and lncRNA play important roles in the response to salinity in rice seedlings and reproductive stages [18,19], the mechanism by which ceRNAs influence salt tolerance and yield in rice has been rarely reported.

‘Yanfeng 47’, a rice variety with strong salt tolerance, is currently the variety most widely planted on saline–alkali land in the Yellow River Delta of China. The present study aims to identify differentially expressed (DE) lncRNAs, miRNAs, and mRNAs in ‘Yanfeng 47’ in response to salinity stress through whole-transcriptome resequencing and to characterize their potential functions in stress response by Gene Ontology (GO) and Kyoto Encyclopedia of Genes and Genomes (KEGG) enrichment analyses. Furthermore, a ceRNA network will be constructed based on the ceRNA hypothesis in response to salinity stress. The results of this study will help in understanding the mechanisms of lncRNA-miRNA-mRNA signaling pathways in rice’s response to salinity and provide a theoretical basis and gene information for future studies on the functions of ceRNA in plant salt tolerance.

## 2. Results

### 2.1. Genome-Wide Identification of lncRNAs, miRNAs, and mRNAs

To comprehensively identify lncRNAs and mRNAs that respond to salt stress during the reproductive stage, strand-specific cDNA libraries were constructed from total RNAs isolated from six samples of rice panicles and sequenced. A total of 573,796,212 clean reads were obtained with an average alignment percentage of 94.75% (Table S1). For small RNA sequencing, a total of 13,557,742 to 18,773,020 reads were obtained from the six sample libraries. After filtering, at least 10,442,897 clean reads were retained for each sample (Table S2).

**Figure 1:**
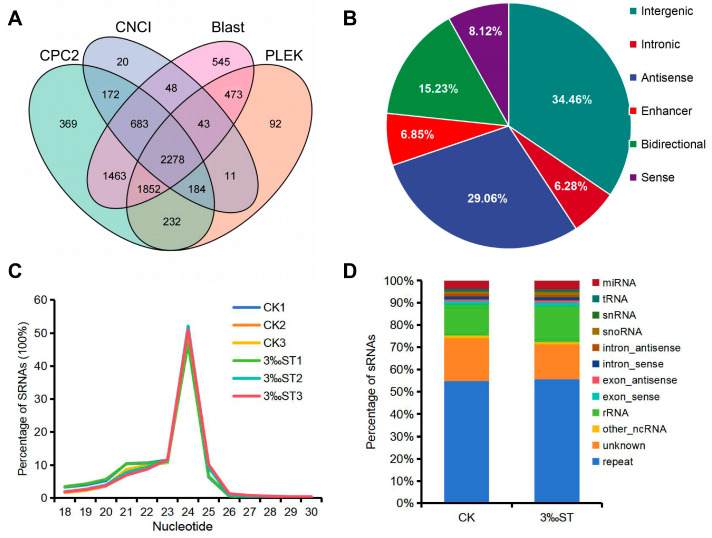
Identification of lncRNAs and small RNAs responsive to salt stress in rice ‘Yanfeng 47’. (**A**) Venn diagram of lncRNAs identified by CPC2, CNCI, BLAST, and PLEK. (**B**) Classification of lncRNAs according to their genomic position and overlap with protein-coding genes. (**C**) Length distribution of small RNAs ranging from 18 to 30 nt. (**D**) Annotation of small RNA types, including rRNA (ribosomal RNA), tRNA (transfer RNA), snRNA (small nuclear RNA), and snoRNA (small nucleolar RNA).

After basic screening, as described in the Section 4, transcripts without coding abilities were further evaluated using CNCI, CPC2, BLAST, and PLEK. Ultimately, 2278 lncRNAs (comprising 235 known and 2043 novel lncRNAs) were identified from the panicle samples using the four methods (Figure 1A). Based on their genomic locations, the lncRNAs were classified as long intergenic non-coding RNAs (785, 34.46%), intronic lncRNAs (143, 6.28%), antisense lncRNAs (662, 29.06%), enhancer lncRNAs (156, 6.85%), bidirectional lncRNAs (347, 15.23%), and sense intronic lncRNAs (185, 8.12%) (Figure 1B). Analysis of the lncRNA characteristics revealed that the length, number of exons, and expression level of the lncRNAs were significantly lower than those of mRNAs (Figure S1A–C).

Screening against the rice genome revealed that the small RNA (sRNA) length in all samples ranged from 18 to 30 nt, with 21 and 24 nt being the most common lengths (Figure 1C). After annotating the small RNA types, we detected 776 miRNAs (738 known and 38 novel). These were distributed among 118 miRNA families based on their miRNA precursors (Figure 1D and Table S3). Among the identified miRNAs, miR812 (25) and miR166 (25) were the most abundant, followed by miR395 (24) and miR2118 (23). The correlation coefficients for abundance among the three biological replicates for each group ranged from 0.986 to 0.998 for lncRNAs, from 0.896 to 0.999 for mRNAs, and from 0.988 to 0.997 for miRNAs (Figure S2), indicating the high quality of the sequencing data.

### 2.2. Analysis of DE-lncRNAs, DE-mRNAs and DE-miRNAs

**Figure 2:**
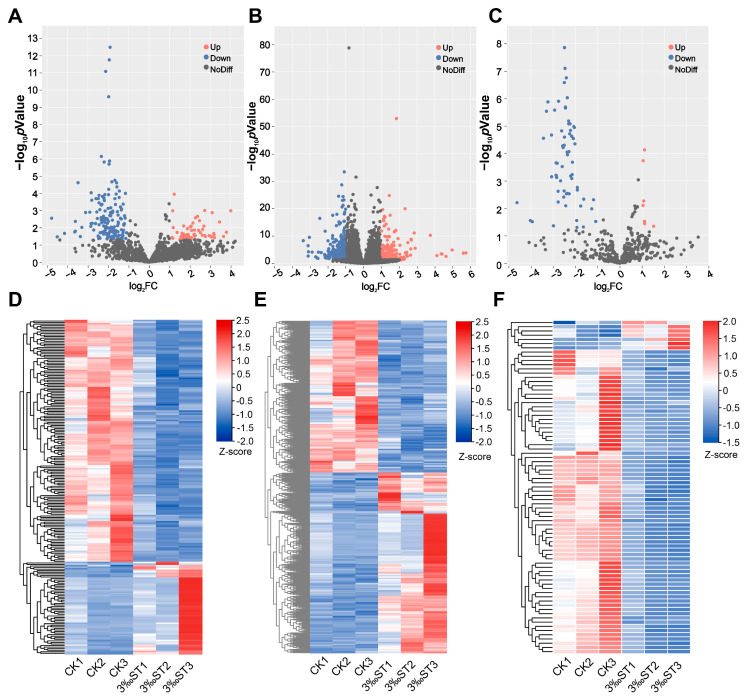
Identification and hierarchical cluster analysis of differentially expressed (DE) lncRNAs, DE-miRNAs, and DE-mRNAs. Volcano plots of DE-lncRNAs (**A**), DE-mRNAs (**B**), and DE-miRNAs (**C**) under salt stress. Hierarchical cluster analysis of the DE-lncRNAs (**D**), DE-mRNAs (**E**), and DE-miRNAs (**F**). The lncRNAs, miRNAs, and mRNAs identified as differentially expressed were used to perform the hierarchical cluster analysis. The bar color indicates the Z-score of the FPKM (fragments per kilobase of transcript per million mapped reads) or TPM (transcripts per kilobase per million mapped reads) values across all samples.

To identify the DE-lncRNAs, DE-mRNAs, and DE-miRNAs in response to salt stress, the normalized expression levels of these RNAs were compared between the salt treatment and control groups. A total of 214 DE-lncRNAs were identified, with 59 upregulated and 155 downregulated lncRNAs, (Table S4). Among the miRNAs, 79 DE-miRNAs were identified, consisting of 7 upregulated and 72 downregulated miRNAs (Table S5). Additionally, 584 DE-mRNAs were identified, with 317 upregulated and 267 downregulated mRNAs (Table S6). These results were visualized in a volcano plot (Figure 2A–C). According to the functional annotations, 71 (12.16%) of the DE-mRNAs were transcription factors. The transcription factor families with the highest number of members were MYB (13), AP2/ERF (10), WRKY (4), and bHLH (4) (Table S6). Some reported genes involved in the salt stress response, such as *OsERF922*, *OsDREB1F*, and *OsSIK2*, were also identified [20,21,22]. A hierarchical cluster analysis was performed on the DE-lncRNAs, DE-mRNAs, and DE-miRNAs with similar expression patterns, showing how their expression changed in response to salt stress (Figure 2D–F). These DE-lncRNAs, DE-miRNAs, and DE-mRNAs provide valuable information for further potential functions.

### 2.3. Analysis of Potential Targets of lncRNAs and miRNAs

To study the roles of salt stress-responsive DE-lncRNAs, potential *cis-* and *trans-*target transcripts were identified. The 214 DE-lncRNAs were predicted to have potential *cis-*regulatory effects on 6156 protein-coding genes (Table S7). Among these lncRNAs, 89.72% targeted more than 20 to 68 transcripts, and eight lncRNAs targeted more than 40 transcripts (Figure S3A). More than 98% of the transcripts corresponded to one to four lncRNAs, and 52 transcripts were predicted to be *cis-*regulated by more than four lncRNAs (Figure S3B). In addition, 773 transcripts were predicted to be trans-regulated by DE-lncRNAs based on the principle of complementary base pairing (Table S7). Among these lncRNAs, 19.72% targeted more than 100 transcripts, and seven transcripts were predicted to be targeted by more than 150 lncRNAs (Figure S3C,D).

To elucidate the biological functions of the DE-miRNAs in response to salt stress, potential target genes, including coding and non-coding genes, were predicted based on almost complete complementarity. A total of 730 coding genes targeted by DE-miRNAs were predicted (Table S8). Almost all miRNAs, except for novel0014 and osa-miR5797, targeted more than one coding gene, and six miRNAs targeted more than 30 coding genes (Figure S3E). Moreover, only 59.18% of protein-coding genes corresponded to a single miRNA, and LOC_Os06g49000 was predicted to be targeted by 10 DE-miRNAs (Figure S3F). Similarly, 643 lncRNAs were identified as non-coding genes for DE-miRNAs (Figure S3G,H). These results indicate the complex regulatory relationships among lncRNAs, miRNAs, and mRNAs.

### 2.4. Functional Enrichment Analysis of DE-mRNAs, DE-miRNA Target Genes and DE-lncRNA Target Genes

**Figure 3:**
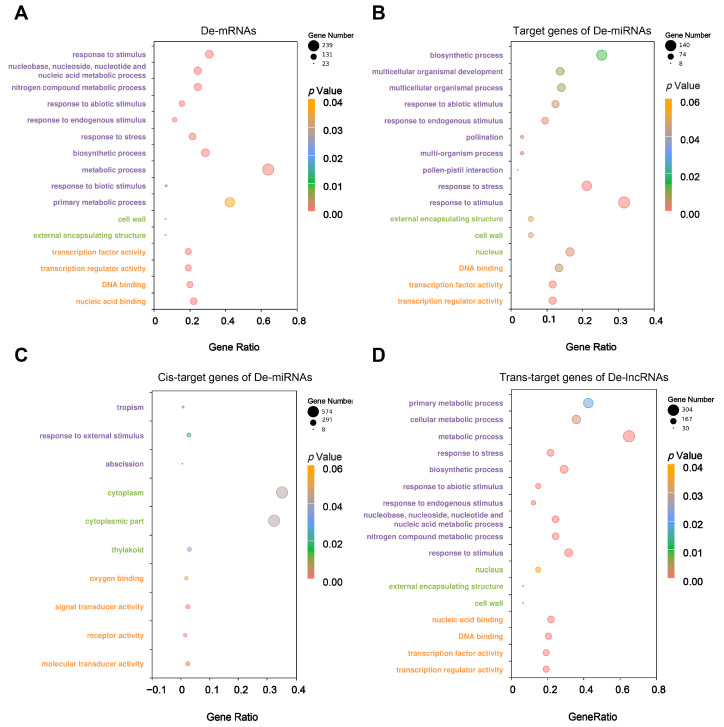
Gene Ontology (GO) analysis of DE-mRNAs, DE-miRNA target genes, and DE-lncRNA target genes. (**A**) Significant GO terms enriched by DE-mRNAs. (**B**) Significant GO terms enriched by target genes of DE-miRNAs. (**C**) Significant GO terms enriched by the *cis*-target genes of DE-lncRNAs. (**D**) Significant GO terms enriched by the *tran*-target genes of DE-lncRNAs. The purple, orange, and green fonts on the y-axis indicate the biological process, molecular function, and cell component categories, respectively. GO terms with *p* < 0.05 were considered significantly enriched.

**Figure 4:**
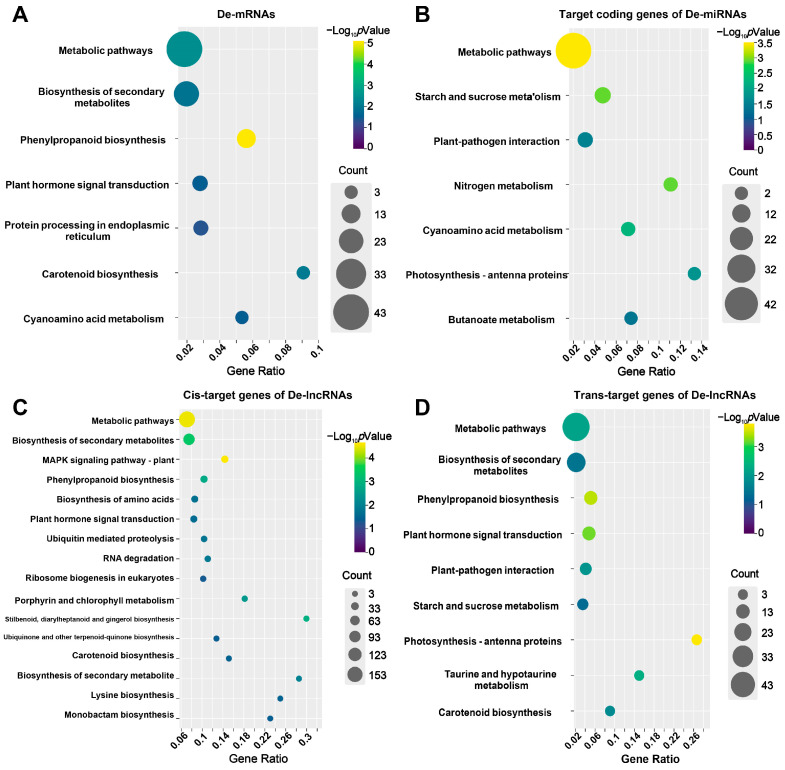
Kyoto Encyclopedia of Genes and Genomes (KEGG) pathway analysis of DE-mRNAs, DE-miRNA target genes, and DE-lncRNA target genes. (**A**) Significant pathways enriched by DE-mRNAs. (**B**) Significant pathways enriched by target genes of DE-miRNAs. (**C**) Significant pathways enriched by the *cis*-target genes of DE-lncRNAs. (**D**) Significant pathways enriched by the *trans*-target genes of DE-lncRNAs. The KEGG pathways with *p* < 0.05 were considered significantly enriched.

GO and KEGG pathway enrichment analyses were used to explore the functions of DE-lncRNAs, DE-miRNAs, and DE-mRNAs in response to salt stress. For DE-mRNAs, 10 biological processes, two cell components, and four molecular functions were significantly enriched. The most highly enriched GO terms in the biological processes, cellular components, and molecular functions categories were “response to stimulus”, “cell wall”, and “transcription factor activity”, respectively (Figure 3A). Seven KEGG pathways, including phenylpropanoid biosynthesis, the metabolic pathway, and carotenoid biosynthesis, were involved in the response to salt stress (Figure 4A). For DE-miRNAs, their target genes were significantly enriched in 16 GO terms and 7 KEGG pathways. The most highly enriched GO terms in the biological processes, cellular components, and molecular functions categories were “biosynthetic process“, “external encapsulating structure“ and “DNA binding“, respectively (Figure 3B). Metabolic pathways, starch and sucrose metabolism, and nitrogen metabolism were the top three significantly enriched pathways in the KEGG analysis (Figure 4B). The GO analysis of the *cis*- and *trans*-target genes of DE-lncRNAs revealed that *trans*-target genes shared similar enriched pathways to DE-mRNAs and targets of DE-miRNAs (Figure 3C,D). The KEGG analysis revealed that five pathways were identical for the DE-mRNAs and the *cis*- and *trans*-target genes of DE-lncRNAs, which comprised the biosynthesis of secondary metabolites, carotenoid biosynthesis, metabolic pathways, phenylpropanoid biosynthesis, and plant hormone signal transduction (Figure 4C,D). These results suggest that the enriched GO and KEGG terms are roughly similar among the three types of RNA. Additionally, pollination, pollen–pistil interaction, the multi-organism process, multicellular organismal development, and the response to abiotic stimulus were specifically noted among the targets of DE-miRNAs (Figure 3B). Signal transducer activity, receptor activity, oxygen binding, and molecular transducer activity were specifically identified among the *cis*-targets of DE-lncRNAs (Figure 3C).

### 2.5. Construction of ceRNA Network Associated with Salt Tolerance

**Figure 5:**
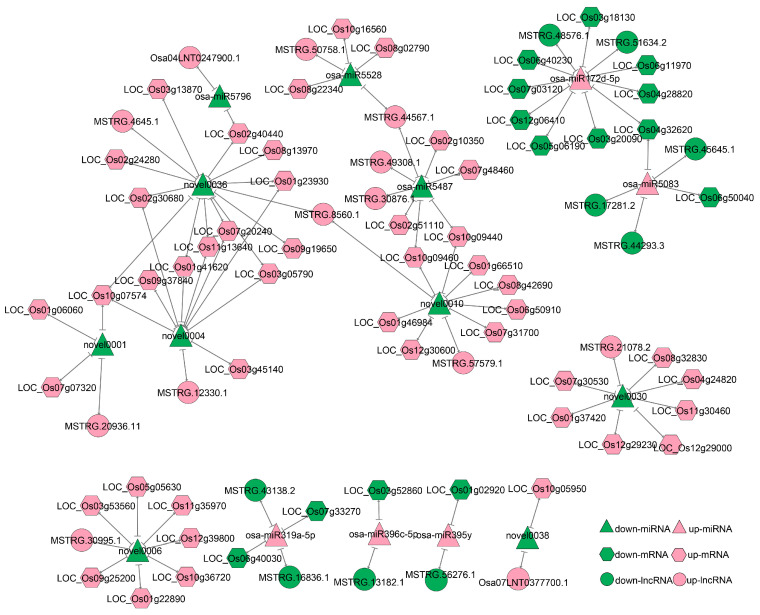
ceRNA network for ‘Yanfeng 47’ rice under salt stress. A panoramic network composed of 22 lncRNAs, 15 miRNAs, and 59 mRNAs. A pink hexagon indicates an upregulated gene, a green hexagon indicates a downregulated gene, a pink triangle indicates an upregulated miRNA, a green triangle indicates a downregulated miRNA, a pink circle indicates an upregulated lncRNA, and a green circle indicates a downregulated lncRNA.

To explore the potential interactions between lncRNAs, mRNAs, and miRNAs in rice under salt stress, we constructed a ceRNA network. First, we predicted the relationships between miRNAs and their target mRNAs or lncRNAs (Table S9). Then, we constructed the ceRNA network under salt stress based on the targeting relationships of miRNA–mRNA and miRNA–lncRNA, as well as the co-expression relationship (r ≥ 0.8, *p* < 0.05) between lncRNA-mRNA regulated by the same miRNA. In total, 95 lncRNA–miRNA–mRNA interactions were included in the ceRNA network, consisting of 15 miRNAs, 22 lncRNAs, and 59 mRNAs (Table S10). We used Cytoscape 3.10.1 to construct a regulatory network diagram (Figure 5).

Two lncRNAs, MSTRG.51634.2 and MSTRG.48576.1, have been identified as potential binders of osa-miR172d-5p, regulating 10 protein-coding genes, including the salt-related gene *LOC_Os03g20090* (*OsMYB2*) [23] and the yield-related gene *LOC_Os06g11970* (*OsMADS63*) [24]. Additionally, three key lncRNAs, MSTRG.30876.1, MSTRG.44567.1, and MSTRG.49308.1, have been found to potentially bind osa-miR5487, regulating the expression of three protein-coding genes. Among these, *LOC_Os07g48460* encodes a stress protein and *LOC_Os02g51110* encodes an aquaporin protein.

**Figure 6:**
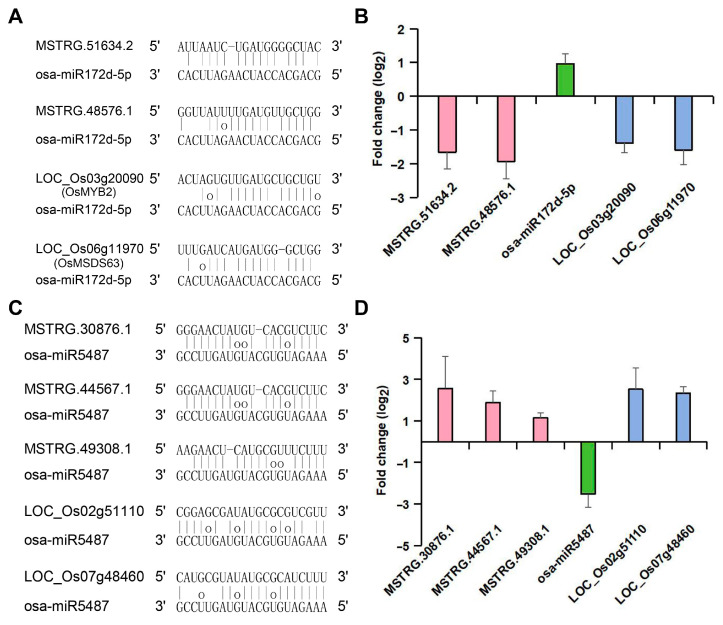
Predicted targets and endogenous target mimics, and expression analysis of osa-miR172d-5p and osa-miR5487. (**A**,**C**) Targets and endogenous target mimics (eTMs) of osa-miR172d-5p and osa-miR5487 in rice cultivar ‘Yanfeng 47′. The predicted base-pairing pattern between the miRNA (osa-miR5487 and osa-miR172d-5p) and their targets and eTMs is shown. A vertical line indicates a Watson–Crick pair, two dots represent a G-U pair, and 0 represents a mismatch. (**B**,**D**) The relative changes in the expression of miRNAs, lncRNAs, and mRNAs [log_2_(fold change) of Illumina reads] between the salt treatment and control libraries. The data are presented as the mean ± SD of three replicates.

The predicted base-pairing pattern between the miRNAs (osa-miR172d-5p and osa-miR5487) and their targets and endogenous target mimics (eTMs) is shown in Figure 6A,C. To increase the reliability of our results, we analyzed the expression levels of osa-miR172d-5p/osa-miR5487 and their targets and eTMs. Under salt stress, the expression of osa-miR172d-5p was upregulated by 2.0 times, while the expression of MSTRG.51634.2, MSTRG.48576.1, *OsMYB2* and *OsMADS63* was downregulated by 3.2, 3.8, 2.6 and 3.0 times, respectively (Figure 6B). Similarity, the expression of miR5487 was downregulated by 5.8 times, while the expression of MSTRG.30876.1, MSTRG.44567.1, MSTRG.49308.1, *LOC_Os07g48460* and *LOC_Os07g48460* was upregulated by 5.9, 3.7, 2.2, 5.0 and 5.8 times, respectively, compared to their respective controls (Figure 6D). Co-expression analysis revealed a significant positive correlation (r > 0.8 and *p* < 0.05) between the targets and the eTMs of osa-miR5487 and osa-miR172d-5p, which is consistent with the ceRNA hypothesis (Table S10).

## 3. Discussion

In recent years, numerous studies have shown that miRNAs and lncRNAs play a role in the response to salt stress at the post-transcriptional level [25]. However, the functions of most lncRNAs and miRNAs are not fully understood. In the present study, a comprehensive transcriptome analysis was conducted on the salt-tolerant rice variety ‘Yanfeng 47’ to identify miRNAs, lncRNAs, and mRNAs responsive to salt stress. By integrating bioinformatic analysis, we investigated the potential interactions between miRNAs and mRNAs, as well as between miRNAs and lncRNAs, and the co-expression of mRNAs and lncRNAs. The construction of a ceRNA network provided a clearer insight into the regulatory mechanisms at the transcriptional level under salt stress.

To combat salinity stress, both coding and non-coding genes undergo extensive changes [18]. In the present study, 584 DE-mRNAs were identified under salt stress, of which 71 were transcription factors (Table S6), highlighting the crucial role of transcription factors in the salt stress response. The expression level of *OsERF922* was notably decreased, consistent with its known role as a negative regulatory factor [21]. Conversely, *OsSIK2* [20] and *OsDREB1F* [22], previously identified as positive regulators of salt tolerance in rice, were significantly upregulated in the present study. A previous study showed that salt stress affects rice quality and yield by regulating the transcription levels of some spikelet formation genes (*LAX1*, *LAX2*, *TAW1* and *OSMADS1*), grain-filling genes (*GIF1*, *GIF2* and *OSNF*-*YB1*), and amylose- and protein-content-associated genes (*Chalk5*, *OsAAP6*, *OsGluA2*) [11,26]. In our study, only *LAX1* [27] was differentially expressed. However, the pollen-development-related gene *bHLH142* [28], quality-related gene *OsSSIIa* [29], spike-development-related gene *GY1* [30], and panicle-development-related gene *OsMFT1* [31] were identified as differentially expressed genes (Table S6). Additionally, the miR156–*IPA1* pathway, which has been predicted to be involved in the salt stress response in rice, was not detected in our study [11]. However, osa-miR1432-5p-*OsA10* was found to potentially affect salt stress tolerance and yield in our study, as miR1432 has been found to regulate yield, drought, and rice blast resistance through various target genes [32,33]. P-type H+-ATPase genes such as *AHA4*, *NtPMA4*, and *MHA2* have also been shown to play crucial roles in salt tolerance [34,35,36]. Therefore, we speculate that the aforementioned differences are caused by differences in the variety background, growth conditions, sampling, and other environmental factors. These findings also reflect the complexity of the mechanism by which salt stress affects yield and quality.

We identified five common GO terms, comprising responses to stress, the biosynthetic process, abiotic stimulus and endogenous stimulus, that were highly enriched among DE-mRNAs, targets of DE-miRNAs, and *trans*-targets of DE-lncRNAs (Figure 3). This result suggests a strong correlation and coordination between lncRNA–miRNA–mRNA interactions in response to salt stress. Secondary metabolites, such as carotenoids, anthocyanins, and flavonoids, play important roles in the response to abiotic stresses, particularly salt stress [37,38]. The present KEGG analysis supports this finding, highlighting the importance of the biosynthesis of secondary metabolites, carotenoid biosynthesis, metabolic pathways, and phenylpropanoid biosynthesis for DE-mRNAs, and *cis*- and *trans*-target genes of DE-lncRNAs (Figure 4). In the current study, we identified pollination (GO:0009856) and pollen–pistil interaction (GO:0009875) as important GO terms for targets of DE-miRNAs, indicating that miRNAs play roles in pollen viability and influence productivity. This is consistent with the view that salinity stress during the reproductive stage negatively impacts pollen viability, the spikelet number, grain formation, and panicle initiation, ultimately limiting crop productivity [39].

Previous reports indicate that lncRNAs can act as ceRNAs to inhibit miRNA function and compete with other targets of miRNAs. Using integrated high-throughput expression data, we constructed a ceRNA network for the salt-tolerant rice ‘Yanfeng 47’ in response to salt stress for the first time. Two lncRNAs (MSTRG.51634.2 and MSTRG.48576.1) were predicted to bind to osa-miR172d-5p to regulate the expression of *OsMYB2* and *OsMADS63,* respectively, affecting salt tolerance and yield. This suggests that ceRNA may simultaneously affect salt tolerance and yield in rice. Additionally, three lncRNAs (MSTRG.30876.1, MSTRG.44567.1, and MSTRG.49308.1) may bind to osa-miR5487 to further regulate the expression of a stress protein (LOC_Os07g48460) and an aquaporin protein (LOC_Os02g51110) in response to salt stress. Base-pairing pattern analysis and expression level analysis between osa-miR172d-5p/osa-miR5487 and their targets and eTMs were used to support the ceRNA network (Figure 6). These results enrich our understanding of how ceRNAs influence salt tolerance and yield in rice and provide genetic resources for the further analysis of salt tolerance mechanisms. Comprehensive investigation is needed in the future to understand how these ceRNAs function under salinity stress.

## 4. Materials and Methods

### 4.1. Plant Material and Salinity Stress Treatment

The salt-tolerant rice variety ‘Yanfeng 47’ was used as the experimental material. The plant was cultivated by the Liaoning Saline-Alkali Land Utilization and Research Institute and stored at the Shandong Academy of Agricultural Sciences in China. The experiments on the salinity stress treatment (3‰ ST) and the non-saline control (CK) were conducted in a saline–alkaline region of Dongying, Shandong Province, China (38°15′ N, 118°50′ E). The growth conditions were described in detail in previous research [11]. Seeds were sown in early May, and then the seedlings were transplanted to an irrigated field. The planting density was 25 cm × 14 cm, with one plant per hill in each block of 3 m^2^. For 3% ST and CK, saline water with a conductivity of 4 dS m^−1^ and fresh water with a pH of 8.0 were irrigated from 7 days after transplantation until harvest, respectively. The saline water was prepared according to the method of Zheng et al. [11]. The salinity and pH of the water were monitored daily and adjusted as necessary. After approximately 40 days of salt treatment, young panicles (0.5 g, approximately 0.5–1 cm in length) from each replicate in 3‰ ST and CK were collected and stored at −80 °C.

### 4.2. RNA Library Preparation and Sequencing

Library construction and RNA sequencing were performed by Novogene Co., Ltd. (Beijing, China). Approximately 1.5 µg of RNA per sample was used to construct a strand-specific library. rRNA was removed using the Ribo-zero™ rRNA Removal Kit (Madison, WI, USA). A total of six libraries (CK1, CK2, CK3, 3% ST1, 3% ST2, and 3% ST3; one library per replicate sample) were constructed. The sequencing library was generated using the NEBNext^®^ Ultra™ Directional RNA Library Prep Kit for Illumina^®^ (Ipswich, MA, USA). The libraries were sequenced on an Illumina NovaSeq 6000 platform (Beijing, China). Small RNA libraries were generated from 1 µg of RNA per sample and were constructed using the NEBNext^®^ Multiplex Small RNA Library Prep Set for Illumina^®^ (Ipswich, MA, USA). The prepared miRNA libraries were sequenced on an Illumina HiSeq 2500/2000 platform, and 50 bp single-end reads were generated (Beijing, China).

### 4.3. Small RNA Analysis and miRNA Identification

The low-quality reads were filtered to obtain clean reads using Trim Galore software (https://github.com/FelixKrueger/TrimGalore (accessed on 22 July 2022)). The filtering standards for low-quality reads refer to previous research by Lu et al. [40]. Next, we obtained unannotated reads containing miRNA by filtering out rRNA, tRNA, snRNA, snoRNA and other ncRNA and repetitive sequences. Then, Bowtie [41] was used to align sequences and obtain the location information in the Nipponbare reference genome (MSU v7.0) (http://rice.plantbiology.msu.edu/cgi-bin/gbrowse/rice (accessed on 5 August 2022)), namely mapped reads. Reads from the reference genome were compared with mature miRNA sequences from miRBase (http://www.mirbase.org (accessed on 22 August 2022)) to identify known miRNAs. miRDeep-P2 [42] was used to obtain the possible precursor sequence by comparing the location information of reads to the reference genome. Novel miRNAs were predicted based on the distribution of reads on the precursor sequence.

### 4.4. lncRNA Analysis

We first performed quality control and trimmed adapters using fastp [43] for each RNA-seq dataset. The filtered high-quality sequencing data were mapped to the Nipponbare reference genome (MSU v7.0) using HISAT2 2.2.1 [44]. The transcripts were assembled using StringTie 2.2.3 [45]. Transcripts with lengths greater than 200 nt and that did not overlap with known coding genes were selected as candidate lncRNAs and further screened using CPC2 1.2 [46], CNCI 2 [47], BLAST 2.9.0 [48] and PLEK1.2 [49] to obtain the non-coding genes.

### 4.5. Differentially Expressed lncRNA, miRNA and mRNA under Salt Stress

The FPKM value for lncRNAs and mRNAs was calculated for each sample using StringTie [45]. Additionally, the TPM value for miRNA was calculated using miRDeep-P2 [42]. The screening criteria for DE-miRNAs, lncRNAs and mRNAs were |log2 (fold change)| ≥ 1 and *p* < 0.05 for treatment and control libraries.

### 4.6. Target Gene Prediction and Functional Annotation

The target genes of DE-lncRNAs were predicted as follows: (1) We searched for coding genes located within 100 kb upstream and 100 kb downstream of the DE-lncRNAs as *cis*-target genes [50]. (2) The *trans*-target genes of DE-lncRNAs were identified using the LncTar program with a cutoff of ndG at −0.2 [51]. TarHunter was used to predict miRNA targets with a cutoff prediction score of seven [52]. GO enrichment analysis and KEGG analysis were performed on the AgriGO website (http://systemsbiology.cau.edu.cn/agriGOv2 (accessed on 16 December 2023)) and the KOBAS website (http://kobas.cbi.pku.edu.cn/kobas3 (accessed on 22 December 2023)), respectively. GO terms or KEGG pathways with *p* < 0.05 were considered significantly enriched.

### 4.7. Co-Expression Analysis and CeRNA Network Construction

Using the matrix data of lncRNAs and mRNAs, we calculated the Pearson correlation coefficients between each DE-mRNA and DE-lncRNA. By combining the mRNA-lncRNA co-expression relationship with the DE-miRNA–DE-mRNA and DE-miRNA–DE-lncRNA regulatory relationships, we established the ceRNA network. According to the mechanism of ceRNA, we were more concerned with the positively correlated expression of DE-lncRNA–DE-mRNA (with r > 0.8 and *p* < 0.05). The lncRNA–miRNA–mRNA complex network was constructed using Cytoscape 3.10.1 [53].

## 5. Conclusions

In conclusion, a total of 214 De-lncRNAs, 79 De-miRNAs, and 584 De-mRNAs were identified in the panicles of the salt-tolerant rice variety ‘Yanfeng 47’ under salinity stress in the present study. The GO and KEGG pathway enrichment analysis provided insights into the roles of miRNAs and lncRNAs in response to salt stress. Based on the ceRNA hypothesis, we constructed a ceRNA regulatory network consisting of 95 lncRNA miRNA mRNA triplets under salinity stress. Among them, osa-miR172d-5p/osa-miR5487 and their targets and eTMs were predicted to be important lncRNA–miRNA–mRNA triplets affecting rice salt tolerance and yield. These results enhance our understanding of the salt tolerance mechanism regulated by miRNAs and lncRNAs in rice, and provide novel genetic resources for future research on salt tolerance.

## Data Availability

The raw data supporting the conclusions of this article will be made available by the authors.

## Supplementary Materials

The supporting information can be downloaded at: https://www.mdpi.com/article/10.3390/ijms25158247/s1.
