# Supplementary material for: Identification and Characterization of miRNAs and lncRNAs Associated with Salinity Stress in Rice Panicles
Source: Int J Mol Sci. 2024 Jul 28;25(15):8247. doi: 10.3390/ijms25158247 (PMC11311799; doi:10.3390/ijms25158247)
Supplement: Supplementary file 1 [file ijms-25-08247-s001.zip › Supplementary Figure.pdf]

## Supplementary Figures

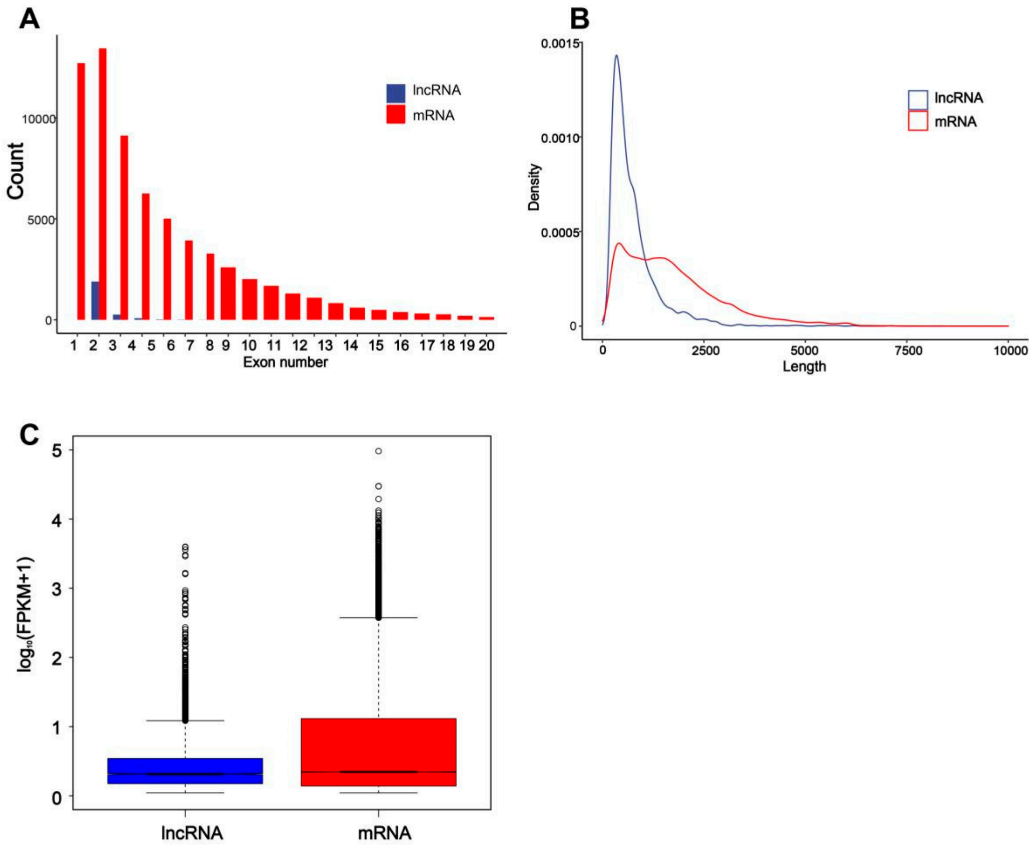


**Figure S1.** Information of the identified lncRNAs and small RNAs. (A) The exon distribution of the identified lncRNAs and mRNAs. (B) The length distribution of the identifed lncRNAs and mRNAs. (C) The comparison of expression levels between lncRNAs and mRNAs.

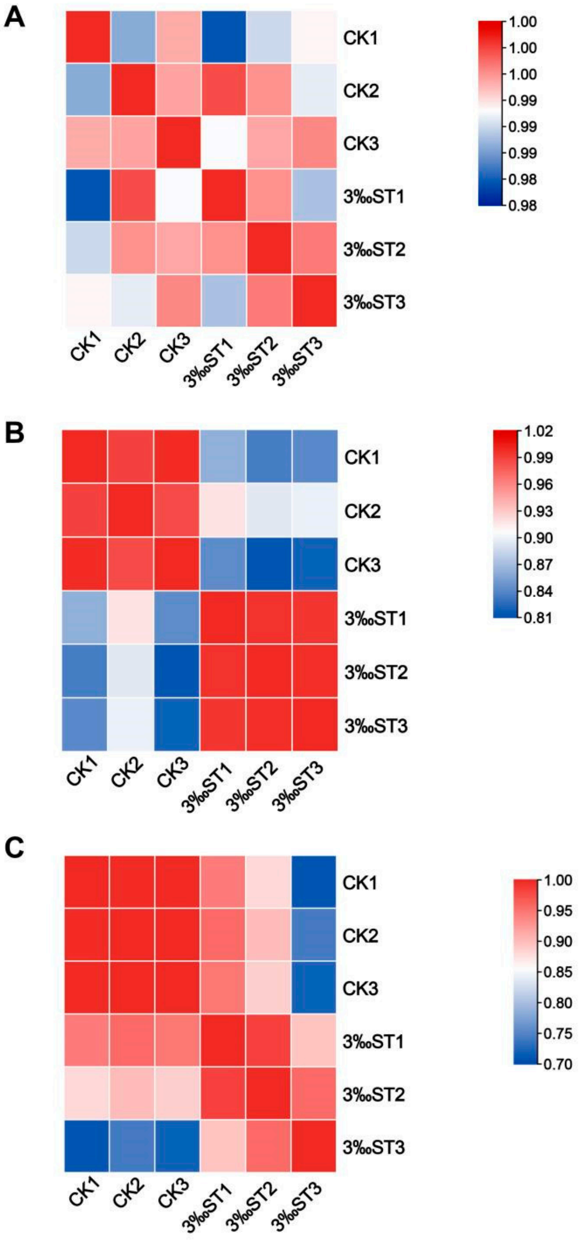


**Figure S2.** Correlation of the miRNA, lncRNA and mRNA abundance between biological replicates. (A) lncRNAs, (B) DE-mRNAs, and (C) DE-miRNAs.

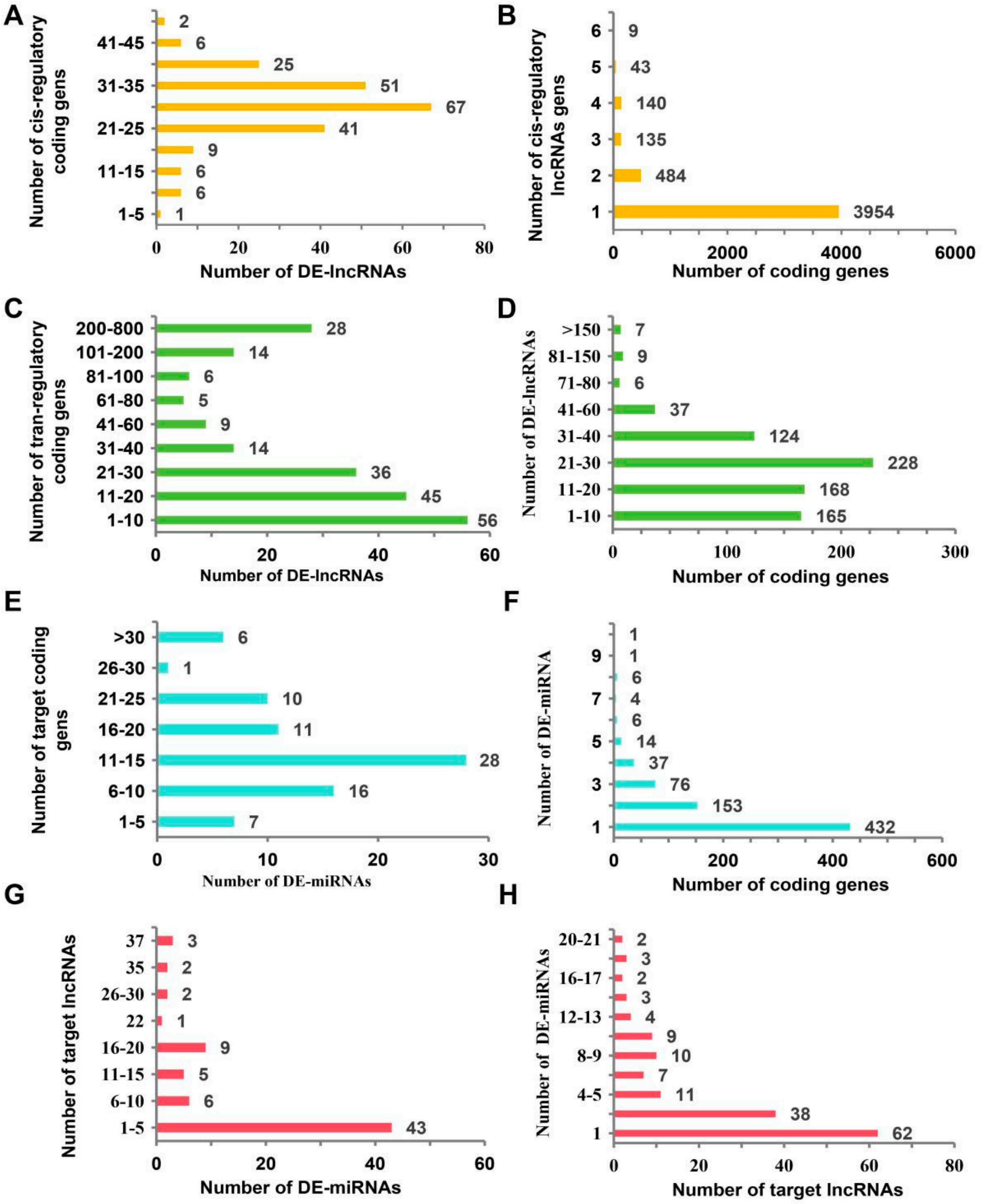


**Figure S3.** Analysis of targets of differentially expressed (DE) lncRNAs and DE-miRNAs. (A) Number of *cis*-target genes regulated by DE-lncRNAs. (B) Number of DE-lncRNAs that have potential *cis*-regulatory effects on protein-coding genes. (C). Number of *trans*-target genes regulated by DE-lncRNAs. (D) Number of DE-lncRNAs that have potential *trans*-regulatory effects on protein-coding genes. (E) Number of target coding genes regulated by DE-miRNAs. (F) Number of DE-miRNAs that have potential effects on coding genes. (G) Number of target lncRNAs regulated by DE-miRNAs. (H) Number of DE-miRNAs that have potential effects on lncRNAs.
